# A Systematic Scoping Review of Participant Descriptor, Diversity and Inclusivity Data Reported in Randomised Studies of Post‐Operative Ileus

**DOI:** 10.1111/ans.70777

**Published:** 2026-06-11

**Authors:** Miriam Khalil, Edward Roberts, David G. Jayne, Stephen J. Chapman

**Affiliations:** ^1^ Leeds Institute of Medical Research, University of Leeds Leeds UK

**Keywords:** general surgery, methods, recovery

## Abstract

**Background:**

Poor reporting of baseline descriptor, diversity and inclusion data is a barrier in surgical research for drawing generalisable conclusions across diverse populations. Post‐operative ileus was used as an exemplar surgical condition to quantify the variation in baseline descriptor, inclusivity and diversity data in randomised controlled trials and to identify targets for improved reporting for baseline descriptors.

**Methods:**

A systematic search of MEDLINE, EMBASE and CENTRAL databases was performed to identify randomised controlled trials (RCTs) in the context of post‐operative ileus after abdominal surgery. Two assessors independently screened studies. Baseline descriptors, including diversity and inclusion descriptors, were extracted and summarised. A narrative synthesis quantified variation and explored targets for improvement.

**Results:**

Of 4887 studies initially identified, 211 met inclusion criteria, and 184 were accessible for inclusion. A total of 92 unique descriptors were identified. The median number of descriptors per RCT was 6 (IQR: 4–8). Characteristics known to predispose patients to ileus, including baseline opioids (*n* = 4/184; 2.2%) and previous surgery (*n* = 38/184; 20.7%), were uncommonly reported. The most common characteristics were age (*n* = 181/184; 98.4%) and one of either sex or gender (*n* = 152/184; 82.6%). A total of 18 (9.8%) RCTs reported participants' ethnic group, described variably as ‘race’ or ‘ethnicity’. No studies reported physical disability.

**Conclusions:**

Substantial heterogeneity exists in the reporting of baseline participant descriptors in the common surgical context of post‐operative ileus. Data relating to diversity and inclusion is seldom reported and difficult to interpret. Homogenising the reporting of these data will improve the comparability and representativeness of future results.

## 1 Introduction

Post‐operative ileus is a common surgical condition experienced by patients undergoing a wide range of abdominal procedures. It is characterised by a cessation of coordinated bowel function, leading to abdominal distension, vomiting and constipation, which may persist for up to 10 days [1]. The impact of post‐operative ileus on patients is highly debilitating since it delays recovery and increases the risk of other serious complications [2]. It is also burdensome for healthcare systems, reducing bed capacity and increasing costs by up to 70% [3]. Accordingly, ileus is recognised as a persisting clinical challenge and a priority for future research by national organisations such as the Association of Coloproctology in Great Britain and Ireland [4].

Across many surgical diseases and conditions, variation in the reporting of baseline participant descriptors is a key challenge. Variation is a barrier to identifying predictors of treatment success and to appraising the external validity of new findings [5]. Poor reporting of inclusion and diversity data is also a limitation for generalising findings across diverse populations and for identifying interventions that may improve outcomes for specific groups. This is particularly true in the context of ileus, for which existing interventional research is limited by unclear signals of efficacy and treatment success [6]. There is a need to homogenise the reporting of these data to improve the comparability and representativeness of future research.

The reporting of descriptor variables can be standardised using a common framework called a core descriptor set. This is a minimum set of data variables, agreed through consensus amongst key stakeholders, which should be reported in all studies of a defined clinical condition. Core descriptor sets already exist for a small selection of diseases, such as Crohn's anal fistula and parastomal hernia [7, 8]. The reporting of diversity and inclusion data, however, remains uncommon and is a challenge which traverses the entire scope of surgical publishing.

Here, the surgical context of post‐operative ileus after abdominal surgery was selected as an exemplar condition. It is a common and broad topic in surgical publishing. The purpose of this review was to quantify the variation in baseline descriptor, inclusivity and diversity data reported in randomised studies on the topic of post‐operative ileus after abdominal surgery. It was expected that this would reveal both context‐specific and broadly generalisable targets across surgical publishing to improve the collection and reporting of baseline descriptors.

## 2 Methods

### 2.1 Study Design

A scoping review was performed using a systematic search strategy in accordance with a predefined protocol. This was selected to enable comprehensive mapping of the heterogeneity of baseline descriptors reported across included studies, a suitable approach to fields where standardisation is lacking. The study aimed to characterise reporting practices and identify gaps to inform future standardisation efforts and did not include a quantitative synthesis. The report described herein is reported in line with the PRISMA Extension for Scoping Reviews [9].

### 2.2 Systematic Search

A search strategy was devised to identify randomised controlled trials (RCTs) in the context of post‐operative ileus after abdominal surgery. A series of keywords was combined using the domains of ‘ileus’, ‘surgery’ and ‘randomised controlled trials’. A full detailed search strategy is provided in Table S1. Two independent investigators (MK and ER) undertook searches of MEDLINE (via Ovid), EMBASE (via Ovid) and CENTRAL on 5 September 2024. Titles, abstracts and full‐text manuscripts were iteratively screened for eligibility, with discrepancies resolved through discussion and consensus with a third investigator (S.J.C.).

### 2.3 Eligibility

All RCTs involving adult patients (≥ 18 years old) receiving any intervention to prevent or reduce ileus after abdominal surgery were eligible. The scope of eligibility was intentionally broad, including studies across all types of abdominal surgery to capture cross‐cutting descriptors (Table S2). Studies published before 2004 were excluded, as were conference abstracts due to the risk of incomplete data. Studies without accessible full text or without available English translation were excluded due to feasibility constraints.

### 2.4 Definitions

RCTs were defined as prospective clinical studies with participants randomised to one of at least two interventional treatment groups. Abdominal surgery was defined as any invasive procedure performed in the abdominopelvic cavity for any clinical indication via an open, laparoscopic, or robotic approach. Studies were considered to be on the subject topic of post‐operative ileus if the primary outcome comprised a clinical, physiological or radiological measure of post‐operative intestinal function or a relevant proxy (such as gastrointestinal quality of life).

### 2.5 Descriptor Variables

Descriptor variables were extracted by two investigators (M.K. and E.R.). The variables of interest were baseline characteristics collected from patients prior to deployment of the study intervention. Data were extracted verbatim from tables and text contained within the published report and relevant appendices. Where appropriate, synonymous descriptors were collapsed and grouped (such as ‘ischaemic heart disease’ and ‘coronary artery disease’), while variables describing distinct characteristics (such as ‘sex’ and ‘gender’) were retained verbatim. In line with previous National Institute of Health and Care Research (NIHR) Diversity Data Reports in the United Kingdom (UK), all included studies were audited against four key variables, comprising age, disability, ethnicity and sex. An explorative assessment for other diversity and inclusion data was also undertaken.

### 2.6 Data and Analysis

Data of interest included manuscript characteristics (journal and year), study characteristics (setting, intervention, outcome and blinding) and procedure characteristics (type of surgery and approach). Descriptive data were expressed using rates, proportions, averages and measures of variance. Subgroup analyses examined variation in the reporting of participant characteristics by clinical context. Studies were classified as gastrointestinal or non‐gastrointestinal. Normality was assessed using the Shapiro–Wilk test. As data were non‐normally distributed, between‐group comparisons were performed using the Mann–Whitney *U*‐test (Wilcoxon rank‐sum), with a two‐tailed *p* < 0.05 considered statistically significant. All data were analysed using IBM SPSS Statistics version 28.0 (IBM, New York, USA).

## 3 Results

### 3.1 Summary of Included Studies

The search identified 4887 studies, of which 211 RCTs were deemed eligible for inclusion; however, only 184 were accessible (Figure 1). The majority of RCTs involved participants undergoing colorectal (*n* = 87/184, 47.3%) and gynaecological (*n* = 25/184, 13.6%) surgery. Most RCTs were single‐centre studies (*n* = 138, 75.0%) reporting small populations (< 100 participants) (*n* = 97, 52.7%). The most common types of intervention were dietary and nutritional (including chewing gum and herbal remedies) (*n* = 79/184; 42.9%), pharmacological (*n* = 69/184; 37.5%) and care pathway interventions (such as goal‐directed fluid management) (*n* = 18/184; 9.8%). A full description of study characteristics is provided in Table 1.

**FIGURE 1:**
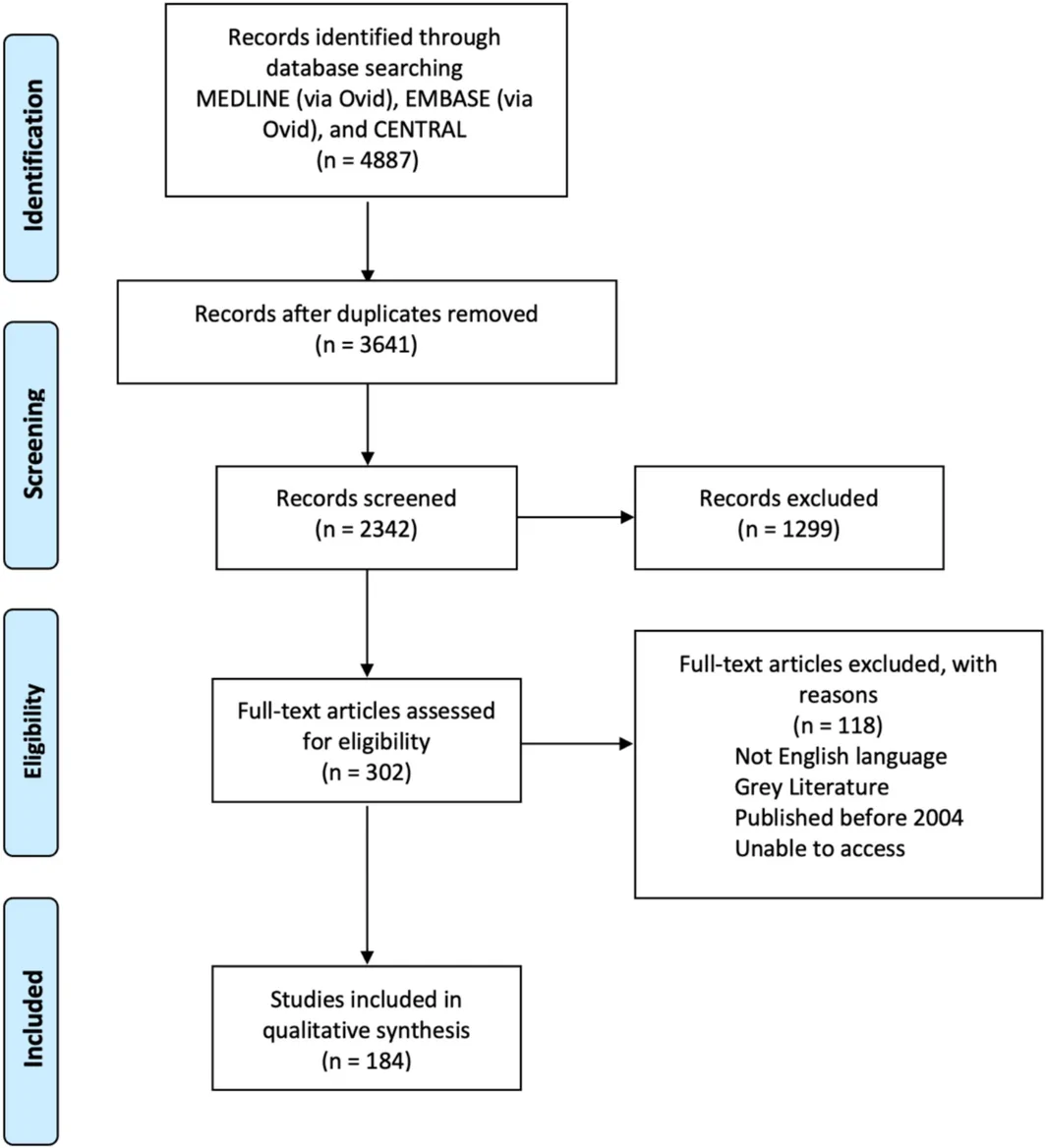
PRISMA diagram.

**TABLE 1:** Characteristics of included studies.

Study characteristics	Frequency (*n*)	%
Year of publication		
2015–2024	117	63.6
2004–2014	67	36.4
Surgery type		
Colorectal surgery	87	47.3
Multiple	30	16.3
Gynaecology and obstetrics	25	13.6
Upper GI surgery	14	7.6
Hepatobiliary surgery	12	6.5
Urology	10	5.4
Other	6	3.3
Number of centres		
Multi‐centre	46	25.0
Single‐centre	138	75.0
Number of arms		
2	151	82.1
3	23	12.5
4+	10	5.4
Number of participants		
< 100	97	52.7
≥ 100	87	47.3
Type of intervention		
Dietary/nutritional	79	42.9
Pharmacological	69	37.5
Care intervention	18	9.8
Device	12	6.5
Surgical/procedural	6	3.3

### 3.2 Descriptor Domains

Across 184 RCTs, a total of 92 unique descriptors were identified (Figure 2). The median number per study was 6 (interquartile range: 4–8), and the most reported in a single study was 18. The extracted descriptors were categorised according to seven conceptual groups, comprising demographic (*n* = 7/92; 7.6%); risk factor (*n* = 9/92; 9.8%); presentation (*n* = 9/92; 9.8%); baseline health (*n* = 42/92; 45.7%); disease‐specific (*n* = 8/92; 8.7%); vital sign (*n* = 6/92; 6.5%) and laboratory (*n* = 11/92; 12.0%) descriptors. Overall, three‐quarters of the extracted descriptors (*n* = 69/92; 75.0%) were reported in fewer than 10 RCTs (*n* = 10/184; 5.4%). A full outline of descriptors and their frequencies is reported in Table 2.

**FIGURE 2:**
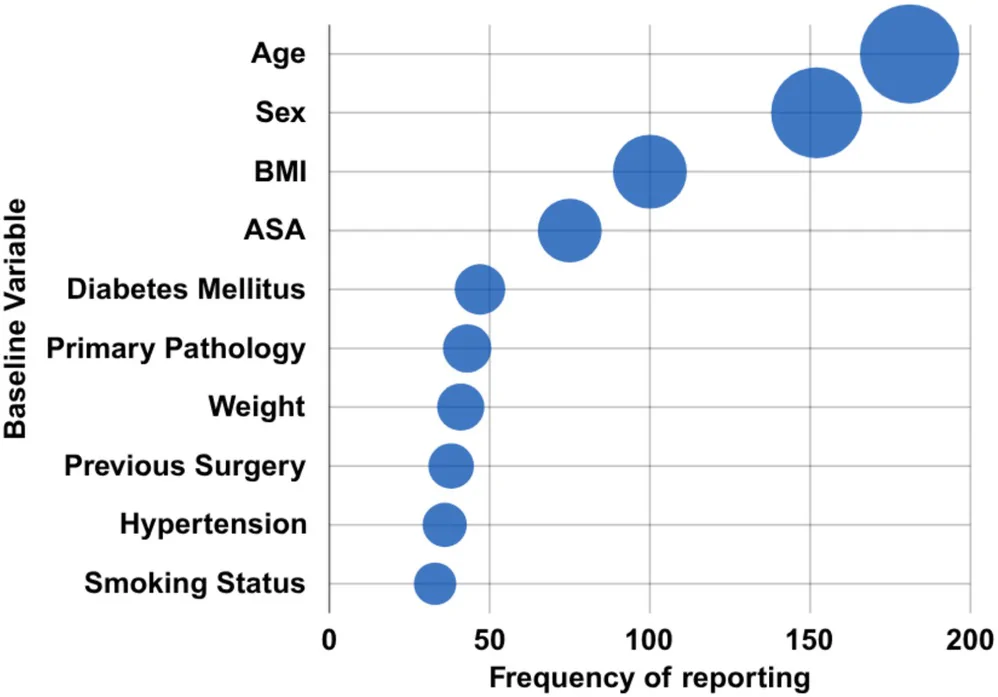
Top 10 most reported descriptors in the context of post‐operative ileus.

**TABLE 2:** Full list of reported baseline descriptors.

Domain	Descriptor	*N*	%
Demographics	Age	181	98.4
	Sex	152	82.6
	Ethnicity	18	9.8
	Education level	8	4.3
	Relationship status	4	2.2
	Residential setting (rural/city)	2	1.1
	Country of birth	1	0.5
Risk factors for ileus	Previous surgery	38	20.7
	Preoperative opioid use	4	2.2
	Preoperative psychoactive drug use	2	1.1
	Preoperative antidepressants use	2	1.1
	History of post‐operative nausea and vomiting	2	1.1
	History of motion sickness	1	0.5
	Pre‐existing stoma	1	0.5
	Preoperative SSRI use	1	0.5
	Preoperative anticholinergic drug use	1	0.5
Presentation factors	Coffee drinker	18	9.8
	Constipation	6	3.2
	Preoperative period without oral intake	4	2.2
	Preoperative use of laxatives	4	2.2
	Preoperative Visual Analogue Scale (pain)	3	1.6
	Preoperative Bristol stool type	2	1.1
	Preoperative bowel movement frequency	1	0.5
	Preoperative Gastrointestinal Symptom Rating Scale	1	0.5
	Pregnant	1	0.5
Measures of baseline health	BMI	100	54.3
	ASA	75	40.8
	Diabetes mellitus	47	25.5
	Weight	41	22.2
	Hypertension	36	19.6
	Smoking status	33	17.9%
	Height	25	13.6
	Cardiovascular comorbidity (undefined)	21	11.4
	Ischaemic heart disease	16	8.7
	COPD	14	7.6
	Respiratory comorbidity (undefined)	10	5.4
	Stroke	10	5.4
	Hyperlipidaemia	7	3.8
	Alcohol use	6	3.2
	Renal comorbidity (undefined)	6	3.2
	Chronic kidney disease	6	3.2
	Nutritional status	5	2.7
	Atrial fibrillation	5	2.7
	Gravidity (pregnancy)	5	2.7
	Performance status	4	2.2
	Hepatic comorbidity (undefined)	4	2.2
	Inflammatory bowel disease	4	2.2
	Gastro‐oesophageal reflux disease	4	2.2
	History of malignant cancers	4	2.2
	Congestive heart failure	3	1.6
	Peripheral artery disease	3	1.6
	Asthma	3	1.6
	Preoperative anticoagulation	3	1.6
	Handgrip strength	2	1.1
	Body SA	2	1.1
	Abdominal aortic aneurysm	2	1.1
	Venous‐thrombo‐embolic event	2	1.1
	Neurological comorbidity (undefined)	2	1.1
	Colitis	2	1.1
	Preoperative antidiabetic therapy	2	1.1
	Preoperative glucocorticoids	2	1.1
	Modified Frailty Index Score	1	0.5
	Menopause status	1	0.5
	Irritable bowel syndrome	1	0.5
	Rheumatoid arthritis	1	0.5
	Preoperative statins	1	0.5
	Parity (pregnancy)	1	0.5
Disease‐specific descriptors	Primary pathology	43	23.4
	Pathology grade/stage	31	16.8
	Neoadjuvant therapy	12	6.5
	Tumour location	10	5.4
	Malignant pathology: preoperative nodal status	9	4.9
	Malignant pathology: preoperative metastatic status	8	4.3
	Tumour perineural invasion	3	1.6
	Tumour size	2	1.1
Vital signs	Baseline heart rate	6	3.2
	Baseline SBP	4	2.2
	Baseline mean arterial pressure	1	0.5
	Baseline MBP	1	0.5
	Baseline DBP	1	0.5
	Hypotension	1	0.5
Laboratory tests	Preoperative haemoglobin	16	8.7
	Preoperative albumin	15	8.2
	Preoperative creatinine	7	3.8
	Preoperative potassium levels	2	1.1
	Preoperative calcium	2	1.1
	Preoperative C‐reactive protein	2	1.1
	Preoperative platelets	2	1.1
	Preoperative haematocrit	1	0.5
	Preoperative white blood cells	1	0.5
	Preoperative total protein	1	0.5
	Preoperative fasting blood glucose	1	0.5

#### 3.2.1 Demographic Descriptors

Of seven demographic descriptors, age was reported most frequently (*n* = 181/184; 98.4%). One of either sex or gender was reported in 152 studies (84.0%), with only 1 study reporting both. Ethnicity was reported in 18 studies (9.78%), education level in 8 studies (4.3%), country of birth in 1 study (0.5%) and residential setting (rural/urban) in 2 studies (1.1%).

#### 3.2.2 Risk Factor Descriptors

Of nine risk factor descriptors for post‐operative ileus, previous surgery (*n* = 38/184; 20.7%) and baseline opioid use (*n* = 4/184; 2.2%) were reported most frequently. All others were reported very infrequently, such as a history of post‐operative nausea and vomiting (*n* = 2/184; 1.1%) and preoperative use of antidepressants (*n* = 2/184; 1.1%) and anticholinergic drugs (*n* = 1/184; 0.5%).

#### 3.2.3 Presentation Descriptors

Of the nine presentation factors, the most frequently reported was a history of coffee consumption (*n* = 18/184; 9.8%). The reporting of other presentation factors was uncommon, with the most frequent being a history of constipation (*n* = 6/184; 3.3%), preoperative use of laxatives (*n* = 4/184; 2.2%) and preoperative duration of time without oral intake (*n* = 4/184; 2.2%).

#### 3.2.4 Baseline Health Descriptors

Baseline health was the most abundantly reported domain (*n* = 42/91). The most frequently reported baseline health descriptors were body mass index (*n* = 100/184; 54.3%), American Society of Anaesthesiologists (ASA) score (*n* = 75/184; 40.8%), history of diabetes mellitus (*n* = 47/184; 25.5%), hypertension (*n* = 36/184; 19.6%) and smoking status (*n* = 33/184; 17.9%).

#### 3.2.5 Disease‐Specific Descriptors

Of eight disease‐specific descriptors, the most frequent were primary pathology (*n* = 43/184; 23.4%) and baseline disease stage (*n* = 31/184; 16.8%). All others were descriptors of malignant disease, such as neoadjuvant therapy (*n* = 12/184; 6.5%), tumour location (*n* = 10/184; 5.4%), preoperative nodal (*n* = 9/184; 4.9%) and metastatic status (*n* = 8/184; 4.3%).

#### 3.2.6 Vital Sign Descriptors

A total of five vital sign descriptors were reported. These included baseline heart rate (*n* = 6/184; 3.3%), systolic blood pressure (*n* = 4/184; 2.2%), diastolic blood pressure (*n* = 1/184; 0.5%) and mean blood pressure (*n* = 1/184; 0.5%), as well as baseline presence of hypotension (*n* = 1/184; 0.5%).

#### 3.2.7 Laboratory Test Descriptors

A total of 11 laboratory descriptors were reported, most frequently comprising preoperative measures of haemoglobin (*n* = 16/184; 8.7%), albumin (*n* = 15/184; 8.2%) and creatinine (*n* = 7/184; 3.8%). Preoperative measures of haematocrit, white cell count, total protein and fasting blood glucose were each reported by only one RCT (*n* = 1/184; 0.5%).

#### 3.2.8 Subgroup Analysis—Gastrointestinal and Non‐Gastrointestinal Contexts

Participant characteristics reported were analysed to investigate variation in reporting between two subgroups of GI and non‐GI (Figure 3). GI studies (*n* = 143) reported the highest number of participant characteristics (median [IQR] = 5 [4–8]), followed by non‐GI studies (*n* = 41) (7 [5–10]). Across 184 RCTs, the number of participant characteristics reported varied significantly (*W* = 2295.5, *p* = 0.0033).

**FIGURE 3:**
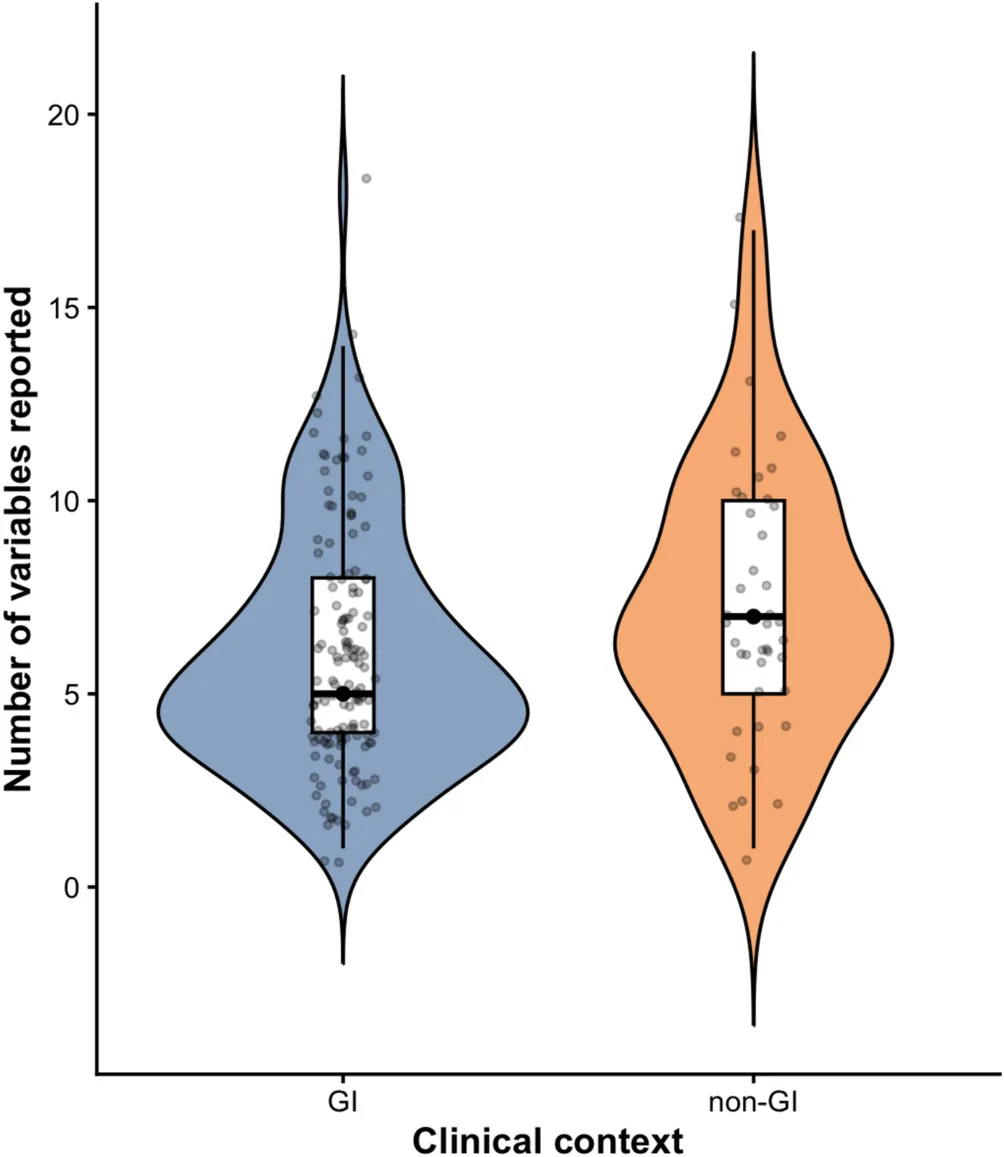
Distribution of the number of baseline descriptors reported across clinical contexts of non‐GI and GI RCTs.

### 3.3 Diversity and Inclusion Data

The reporting of diversity and inclusion data was highly variable. The most frequently reported characteristics were age (*n* = 181/184; 98.4%) and one of either sex or gender (*n* = 152/184; 82.6%). Specifically, ‘male/female’ was reported as ‘sex’ in 80 studies (52.6%) and ‘gender’ in 47 RCTs (30.9%). One RCT (0.67%) reported both concurrently. Only 18 RCTs (9.8%) reported ethnicity or race. This was described variably as ‘race’ (*n* = 9/18; 50%), ‘race/ethnicity’ (*n* = 2/18; 11.1%) and ‘ethnicity’ (*n* = 2/18; 11.1%), with the remainder (*n* = 5/18; 27.8%) not defining a specific label. Common response categories were ‘White’ (*n* = 15/18; 83.3%), ‘Asian’ (*n* = 10/18; 66.7%), ‘Black’ (*n* = 9/18; 50%), ‘Other’ (*n* = 8/18; 44.4%) and ‘Hispanic’ (*n* = 7/18; 38.9%). In three studies (16.7%), Black participants were described as ‘African–American’ with no further clarity for other Black groups. No studies reported the presence or absence of mixed groups. No studies reported descriptors relating to baseline physical disability. Other relevant social descriptors comprised education level (*n* = 6/184; 3.3%), reported mostly using the categories ‘primary’, ‘secondary’ and ‘tertiary’ education, and relationship status (*n* = 2/184; 1.1%), reported by both as ‘single’ or ‘married’.

## 4 Discussion

The results of this review revealed substantial heterogeneity in the reporting of baseline descriptors in RCTs in the context of post‐operative ileus. A total of 92 unique descriptors were identified across 184 RCTs, with a median of only six reported per study. Notably, three‐quarters of all descriptors were reported in fewer than 10 RCTs. Baseline clinical characteristics known to predispose patients to an increased risk of ileus, including opioids and previous surgery, were rarely reported. Most diversity and inclusion‐related descriptors were scarcely documented, with only age and sex being routinely reported across most RCTs.

A lack of consistency in how we report baseline population and disease descriptors is a key challenge for surgical research [6]. Variation in reporting is problematic for drawing comparisons between studies and for appraising their external validity. This creates barriers for evidence synthesis, ultimately reducing our ability to evaluate and implement research reliably into practice. In the last 5 years, core descriptor sets for studies of Crohn's anal fistula and parastomal hernia have been developed in light of significant variation identified in previous literature [7, 8]. These are now used to improve the value of research through improved reporting. As shown by the present data, research in the context of post‐operative ileus is also challenged by variation, and the development of a core descriptor set is similarly justified. In prognostic research, this would facilitate progression through the PROGress RESearch Strategy (PROGRESS) framework, concerned with improving the quality of prognosis‐related studies. Specifically, facilitating recognition of population‐level risk factors for ileus through to stratification of patients based on modifiable and non‐modifiable predictors. In interventional research, it would enable a robust assessment of treatment efficacy across sub‐populations, improving our ability to personalise the management of ileus.

In many areas of health research, descriptions of inclusivity and diversity within study populations are underreported, driving ethnic, racial and social disparities in healthcare. Poor representation of diverse populations in research limits the generalisability of study findings as well as our ability to identify interventions that may improve outcomes for specific groups. The findings of the present work revealed that diversity‐ and inclusion‐related descriptors in the context of post‐operative ileus were commonly underreported. While sex was reported frequently, ethnicity and race were reported in less than 10% of studies, with physical disability not reported at all. Descriptors of sex and gender were used interchangeably to describe males and females in a similar way as race and ethnicity were used to describe participants' ethnic backgrounds. The interchangeable use of these terms reflects a lack of standardisation that limits transparency and hinders the interpretation of inclusion data. Incorporating diversity and inclusion descriptors in core descriptor frameworks could help us to better understand clinical outcomes and to evaluate the impact of interventions for specific communities.

A key strength of this review is its broad scope, including RCTs from a range of surgical contexts to capture a broad scope of descriptors which may be relevant across abdominal surgery settings. This is important and unique since the development of previous core descriptor sets focused on diseases within a defined scope of practice. The present work necessarily crosses multiple fields of surgery within which post‐operative ileus exists as a common complication. Ensuring that all of these descriptors are considered is important for developing a comprehensive and inclusive descriptor set for ileus after abdominal surgery. Limitations are also recognised. The exclusion of studies published in non‐English languages without an accompanying translation, as well as those that were not accessible in full text, represents an important limitation. This limitation is particularly relevant in the context of diversity and inclusion, as it may disproportionately exclude studies conducted in non‐English‐speaking regions. Reporting practices for demographic and inclusion‐related variables may vary internationally, and therefore the present findings could reflect, in part, a Western‐centric evidence base. This raises the consideration that diversity reporting could be under‐represented or differently conceptualised in studies excluded, which cannot be captured by this analysis. Nevertheless, the need for a step‐change to improve inclusion and diversity reporting in this body of literature, as illustrated by the current results, still remains.

This review demonstrates significant heterogeneity in the reporting of baseline participant descriptors in RCTs in the context of post‐operative ileus. In particular, descriptors describing the diversity and inclusivity of study populations were wholly underreported. The findings provide an empirical basis for the development of a consensus‐driven core descriptor set of baseline participant descriptors to be reported in RCTs in the context of post‐operative ileus. The next phase of this work will involve structured consensus methodology, with a Delphi process engaging clinicians, trialists, and patients, to define a minimum reporting standard. Importantly, the present review identifies specific data gaps to inform this process, including the inconsistent reporting of known clinical factors (e.g., prior surgery and opioid use) and the near absence of standardised diversity‐related descriptors. Establishing this consensus‐driven minimum dataset will allow more consistent reporting and enable more meaningful comparison across trials, as well as supporting subgroup analyses aimed at assessing outcomes across different patient sub‐populations.

## Funding

The work is supported by a Royal College of Surgeons of England Pump Priming Award. In addition, D.G.J. is supported in part by the National Institute for Health and Care Research (NIHR213331, NIHR205280) and Bowel Cancer UK (18SC0001). S.J.C. is supported by the National Institute for Health and Care Research (NIHR CL‐2022‐02‐05). The views are those of the authors and not those of the NHS or Bowel Cancer UK.

## Ethics Statement

The authors have nothing to report.

## Consent

The authors have nothing to report.

## Conflicts of Interest

The authors declare no conflicts of interest.

## Supporting information


**Table S1:** Search strategy.
**Table S2:** List of included RCTs.

## Data Availability

The data that support the findings of this study are available from the corresponding author upon reasonable request.
