# Supplementary material for: A Systematic Scoping Review of Participant Descriptor, Diversity and Inclusivity Data Reported in Randomised Studies of Post‐Operative Ileus
Source: ANZ J Surg. 2026 Jun 11;96(10):3084–92. doi: 10.1111/ans.70777 (PMC13724786; doi:10.1111/ans.70777)
Supplement: Supplementary file 1 [file ANS-96-3084-s001.docx]

**SUPPLEMENTARY MATERIAL**

**Table S1 –** Search Strategy

| Ileus | 1. Exp Ileus/ 2. Exp gastrointestinal motility/ 3. Exp intestinal pseudo obstruction/ 4. Exp colonic pseudo obstruction/ 5. Ileus.ti,ab 6. Gastrointestinal adj1 recovery.ti,ab 7. Bowel adj1 function.ti.av 8. Or/1-7 |
| --- | --- |
| Surgery | 9. Exp Specialties, Surgical/ 10. surg$.tw. 11. operat$.tw. 12. intervention$.tw. 13. procedur$.tw. 14. resect$.tw. 15. Or/9-14 |
| Randomised Trials | 16. randomized controlled trial.pt. 17. controlled clinical trial.pt. 18. randomized controlled trials.sh. 19. random allocation.sh. 20. double blind method.sh. 21. single-blind method.sh. 22. clinical trial.pt. 23. exp clinical trials/ 24. (clin$ adj25 trial$).ti,ab. 25. ((singl$ or doubl$ or trebl$ or tripl$) adj25 (blind$ or mask$)).ti,ab. 26. placebos.sh. 27. placebo$.ti,ab. 28. random$.ti,ab. 29. research design.sh. 30. comparative study.sh. 31. Exp evaluation studies/ 32. follow up studies.sh. 33. prospective studies.sh. 34. (control$ or prospective$ or volunteer$).ti,ab. 35. Exp animals/not human/ 36. or/16-34 37. 36 NOT 35 |
| Combination | 38. 8 AND 15 and 37 39. LIMIT (2004-2024) |

**Table S2 – List of included RCTs**

| **Title** | **Authors** | **Year** |
| --- | --- | --- |
| Effects of neuromuscular block reversal with neostigmine/glycopyrrolate versus sugammadex on bowel motility recovery after laparoscopic colorectal surgery: A randomized controlled trial. | Choi et al | 2024 |
| Effects of patient-based self-Assessed fatigue intervention on early postoperative ambulation following gynaecological oncology surgery: A randomised controlled non-inferiority trial. | Du et al | 2023 |
| Surgical effect and gastrointestinal functional recovery of laparoscopic-guided total mesorectal excision in patients with rectal cancer. | Jiang et al | 2024 |
| Bifidobacterium animalis subsp. lactis LPL-RH improves postoperative gastrointestinal symptoms and nutrition indexes by regulating the gut microbiota in patients with valvular heart disease: a randomized controlled trial. | Lan et al | 2024 |
| The effect of Cuminum cyminum on the return of bowel motility after abdominal surgery: a triple-blind randomized clinical trial. | Amin et al | 2024 |
| Effects of transcutaneous electrical nerve stimulation on recovery of gastrointestinal motility after laparotomy: A randomized controlled trial. | Karthik et al | 2024 |
| Perioperative liberal drinking management promotes postoperative gastrointestinal function recovery after gynecological laparoscopic surgery: A randomized controlled trial. | Wang et al | 2024 |
| The Effect of Preoperative Fiber on Postoperative Bowel Function After Pelvic Reconstructive Surgery: A Randomized Controlled Trial. | Maheshwari et al | 2022 |
| Safety of early oral feeding after total laparoscopic radical gastrectomy for gastric cancer (SOFTLY-1): A single-center randomized controlled trial. | Wang et al | 2019 |
| Effect of chewing gum in reducing postoperative ileus after gastroduodenal perforation peritonitis surgery: A prospective randomised controlled trial. | Muwel et al | 2024 |
| Effect of Paula exercise method and Gum Chewing on Post Cesarean Ileus: A randomized controlled clinical trial. [Persian] | Zargani et al | 2024 |
| Effectiveness of Acupressure on Post-Cesarean Bowel Function: A Randomized Controlled Trial. | Sokmen et al | 2024 |
| Comparative analysis of low versus normal pressure pneumoperitoneum in laparoscopic gynecological surgeries: a randomized control trial. | Gaurav et al | 2024 |
| A randomized controlled study to assess the effect of mosapride citrate on intestinal recovery following gastrectomy. | Jun et al | 2024 |
| Prophylactic Intra-abdominal Drainage is Associated With Lower Postoperative Complications in Patients With Crohn's Disease: A Randomized Controlled Trial. | Duan et al | 2024 |
| Effectiveness of ERAS Protocol in Gynaecological Surgeries: Experience at a Tertiary Care Centre in India. | Suresh et al | 2024 |
| Gastrografin may reduce time to oral diet in prolonged post-operative ileus: a pooled analysis of two randomized trials. | Milne et al | 2018 |
| Effects of intravenous dexmedetomidine versus lidocaine on postoperative pain, analgesic consumption and functional recovery after abdominal gynecological surgery: A randomized placebo-controlled double blind study. | Rekatsina et al | 2021 |
| Effect of abdominal hot pack application on gastrointestinal motility recovery after comprehensive gynecologic staging surgery. | Gungorduk et al | 2024 |
| Effect and mechanism of needleless transcutaneous neuromodulation on gastrointestinal function after pancreaticoduodenectomy. | Wu et al | 2024 |
| Comparison of three different prophylactic treatments for postoperative nausea and vomiting after total joint arthroplasty under general anesthesia: a randomized clinical trial. | Xie et al | 2024 |
| To Evaluate Early Feed vs Late Feed in Post-lower-segment Cesarean Section Patients at a Tertiary Care Center: A Randomized Controlled Study. | Choudhari et al | 2023 |
| Rectal misoprostol after cesarean delivery: Does it affect recovery of bowel functions? A prospective randomized trial. | Aref N.K. | 2020 |
| Prospective Randomized Trial of Immediate Postoperative Use of Regular Diet Versus Clear Liquid Diet in Major Colorectal Surgery. | Yao et al | 2023 |
| COMPARATIVE STUDY OF OUTCOME OF PERITONEAL LAVAGE USING SALINE, METRONIDAZOLE AND BETADINE SOLUTION IN PERITONITIS. | Singh et al | 2023 |
| Coffee administration to promote return of bowel function after small bowel resection: A randomized, controlled trial. | Holland et al | 2023 |
| Does coffee affect bowel recovery following minimally invasive colorectal operations? A three-armed randomized controlled trial. | Nasseri et al | 2023 |
| Application effect of gastrointestinal bundle nursing on the protection of gastrointestinal function in patients with gastric cancer. | Wei et al | 2023 |
| Effect of Chewing Gum on Recovery of Intestinal Function after Caesarean Section. | Singh et al. | 2023 |
| Enhanced recovery program after laparoscopic colorectal surgery during the era of COVID-19: a randomized controlled trial. | Negm et al | 2023 |
| Effect of prucalopride to improve time to gut function recovery following elective colorectal surgery: Randomized clinical trial. | Milne et al. | 2022 |
| Effect of non-mechanical bowel preparation on postoperative gastrointestinal recovery following surgery on malignant gynecological tumors: A randomized controlled trial. | Wang et al | 2023 |
| The effect of chamomile on flatulence after the laparoscopic cholecystectomy: A randomized triple-blind placebo-controlled clinical trial. | Salimi et al | 2023 |
| Effect of remote ischemic preconditioning in patients undergoing laparoscopic colorectal cancer surgery: a randomized controlled trial. | Yang et al | 2023 |
| Low-intensity transcutaneous auricular vagus nerve stimulation reduces postoperative ileus after laparoscopic radical resection of colorectal cancer: a randomized controlled trial. | Ru et al | 2023 |
| CONSORT-epidural dexmedetomidine improves gastrointestinal motility after laparoscopic colonic resection compared with morphine. | Wan et al | 2018 |
| Effect of lidocaine or dexmedetomidine on postoperative recovery in elder patients undergoing colorectal surgery. | Qin et al | 2023 |
| Effect of lidocaine or dexmedetomidine on postoperative recovery in elder patients undergoing colorectal surgery. | Baumgartner et al | 2022 |
| Bowel stimulation before loop ileostomy closure to reduce postoperative ileus: a multicenter, single-blinded, randomized controlled trial. | Garfinkle et al | 2023 |
| Enhanced recovery after surgery (ERAS) protocols in patients undergoing radical cystectomy with ileal urinary diversions: A randomized controlled trial. | Vlad et al | 2020 |
| Effect of Intraoperative Dexmedetomidine Infusion on Postoperative Bowel Movements in Patients Undergoing Laparoscopic Gastrectomy A Prospective, Randomized, Placebo-Controlled Study. | Cho et al | 2015 |
| Association of gum chewing with early gastrointestinal recovery in single-port laparoscopic gynecologic surgery. | Yin et al | 2023 |
| The preventive effect of oral 76% Meglumine Diatrizoate for the postoperative ileus. | Sun et al | 2023 |
| Effect of early physical therapy interventions on post-operative ileus following abdominal hysterectomy. | Zia et al | 2023 |
| Hand-sewn direct repair versus resection and hand-sewn anastomosis techniques for the reversal of diverting loop ileostomy after lower anterior rectal resection surgery: A randomized clinical trial. | Keramati et al | 2023 |
| Electroacupuncture vs Sham Electroacupuncture in the Treatment of Postoperative Ileus after Laparoscopic Surgery for Colorectal Cancer: A Multicenter, Randomized Clinical Trial. | Wang et al | 2023 |
| The orange-III study: the use of preoperative laxatives prior to liver surgery in an enhanced recovery programme, a randomized controlled trial. | van Woerden et al | 2022 |
| The effects of fentanyl, oxycodone, and butorphanol on gastrointestinal function in patients undergoing laparoscopic hysterectomy: a prospective, double-blind, randomized controlled trial. | Guo et al | 2022 |
| Effects of Nalbuphine on Gastrointestinal Function in Post-Operative Critical Ill Patients Admitted to the ICU: A Multicenter Randomized Controlled Trial. | Yan et al | 2022 |
| Bowel function after laparoscopic right hemicolectomy: a randomized controlled trial comparing intracorporeal anastomosis and extracorporeal anastomosis. | Malczak et al | 2022 |
| Effects of preoperative walking on bowel function recovery for patients undergoing gynecological malignancy laparoscopy. | Xia et al | 2022 |
| The effect of coffee consumption on improving bowel function after abdominal hysterectomy compared to routine diet: a randomized double-blind clinical-trial. [Persian] | Ghomian et al | 2022 |
| The effect of chewing gum on bowel function postoperatively in patients with total laparoscopic hysterectomy: a randomised controlled trial. | Kadirogullari et al | 2022 |
| A randomized controlled trial of the effect of ramosetron on postoperative restoration of bowel motility after gynecologic laparoscopic surgery. | Kang et al | 2022 |
| Effect of acupuncture on postoperative ileus after laparoscopic elective colorectal surgery: A prospective, randomised, controlled trial. | Yang et al | 2022 |
| Effect of Wuda granule on gastrointestinal function recovery after laparoscopic intestinal resection: a randomized-controlled trial. | Zeng et al | 2022 |
| Early enteral feeding is beneficial for patients after pelvic exenteration surgery: A randomized controlled trial. | Hogan et al | 2022 |
| Preoperative carbohydrate loading with individualized supplemental insulin in diabetic patients undergoing gastrointestinal surgery: A randomized trial. | Li et al | 2022 |
| Effects of liberal oral intake on postoperative outcomes after elective laparoscopic gynaecological surgery: A randomised controlled clinical trial. | Bao et al | 2022 |
| Effects of Gum Chewing on Recovery From Postoperative Ileus: A Randomized Clinical Trail. | Hsu and Szu | 2022 |
| Balanced Opioid-free Anesthesia with Dexmedetomidine versus Balanced Anesthesia with Remifentanil for Major or Intermediate Noncardiac Surgery: The Postoperative and Opioid-free Anesthesia (POFA) Randomized Clinical Trial. | Beloeil et al | 2021 |
| Ultrasound delivery of Chinese rhubarb promotes early recovery of gastrointestinal function after gastrectomy: a prospective randomized controlled study. | Jia et al | 2021 |
| Transcutaneous electrical acupoint stimulation applied in lower limbs decreases the incidence of paralytic ileus after colorectal surgery: A multicenter randomized controlled trial. | Gao et al | 2021 |
| Impact of drinking Chinese green tea on postoperative short outcomes for gastric cancer: a randomized controlled trial. | Liu et al | 2021 |
| The impact of low pressure pneumoperitoneum in robotic assisted radical prostatectomy: a prospective, randomized, double blinded trial. | Rohloff et al | 2021 |
| The effect of intraoperative fluid management according to stroke volume variation on postoperative bowel function recovery in colorectal cancer surgery. | Lee et al | 2021 |
| Postoperative ileus after stimulation with probiotics before ileostomy closure. | Rodriguez-Padilla et al | 2021 |
| Effect of Intraoperative Dexmedetomidine on Recovery of Gastrointestinal Function after Abdominal Surgery in Older Adults: A Randomized Clinical Trial. | Lu et al | 2021 |
| Randomized Controlled Trial: Perioperative Dexamethasone Reduces Excessive Postoperative Inflammatory Response and Ileus after Surgery for Inflammatory Bowel Disease. | Zhang et al | 2021 |
| Impact of intravenous dexmedetomidine on postoperative bowel movement recovery after laparoscopic nephrectomy: A consort-prospective, randomized, controlled trial. | Huang et al | 2021 |
| The role of tibial nerve stimulation for enhanced postoperative recovery after colorectal surgery: a double-blind, parallel-group, randomized controlled trial. | Martellucci et al | 2021 |
| Effects of acupressure, gum chewing and coffee consumption on the gastrointestinal system after caesarean section under spinal anaesthesia. | Kanza Gul et al | 2021 |
| Does caffeine enhance bowel recovery after elective colorectal resection? A prospective double-blinded randomized clinical trial. | Parnasa et al | 2021 |
| Effects of lactobacillus rhamnosus gg on early postoperative outcome after pylorus-preserving pancreatoduodenectomy: A randomized trial. | Folwarski et al | 2021 |
| Noninvasive vagus nerve stimulation to reduce ileus after major colorectal surgery: early development study. | Chapman et al | 2021 |
| Effects of probiotics on bowel function restoration following ileostomy closure in rectal cancer patients: a randomized controlled trial. | Yoon et al | 2021 |
| Influence of preoperative enema application on the return of gastrointestinal function in elective Cesarean sections: a randomized controlled trial. | Ertas et al | 2021 |
| Impact of STIMUlant and osmotic LAXatives (STIMULAX trial) on gastrointestinal recovery after colorectal surgery: randomized clinical trial. | Dudi-Venkata et al | 2021 |
| Systemic versus Oral and Systemic Antibiotic Prophylaxis (SOAP) study in colorectal surgery: prospective randomized multicentre trial. | Papp et al | 2021 |
| Role of Chewing Gum in Reducing Postoperative Ileus after Reversal of Ileostomy: A Randomized Controlled Trial. | Bhatti et al | 2021 |
| Are Paula method exercises effective for gastrointestinal reactivation post-elective cesarean delivery? Randomized controlled trial. | Liebergall-Wischnitzer et al | 2021 |
| Balanced Opioid-free Anesthesia with Dexmedetomidine versus Balanced Anesthesia with Remifentanil for Major or Intermediate Noncardiac Surgery. | Beloeil et al | 2021 |
| ELECTROACUPUNCTURE COMBINED WITH FAST-TRACK PERIOPERATIVE PROGRAM FOR REDUCING DURATION OF POSTOPERATIVE ILEUS AND HOSPITAL STAY AFTER LAPAROSCOPIC COLORECTAL SURGERY: A RANDOMIZED CONTROLLED TRIAL. | Ng et al | 2021 |
| Effect of Gum Chewing on Recovery After Surgery for Colorectal Surgery Patients: A Randomized Controlled Trial. | Duluklu and Senol | 2020 |
| Can coffee consumption be used to accelerate the recovery of bowel function after cesarean section? Randomized prospective trial. | Bozkurt et al | 2020 |
| Effect of Chewing Gum on Gastrointestinal Function Recovery After Surgery of Gynecological Cancer Patients at Rajavithi Hospital: A Randomized Controlled Trial. | Nanthiphatthanachai and Insin | 2020 |
| Heated fennel therapy promotes the recovery of gastrointestinal function in patients after complex abdominal surgery: A single-center prospective randomized controlled trial in China. | Chen et al | 2020 |
| Effects of Daikenchuto on postoperative gastrointestinal motility in colorectal carcinoma patients with abdominal pain and distension: a prospective, randomized trial. | Wakasugi et al | 2020 |
| Gastrointestinal Complications after Pancreatoduodenectomy with Epidural vs Patient-Controlled Intravenous Analgesia: A Randomized Clinical Trial. | Klotz et al | 2020 |
| Coffee consumption for recovery of intestinal function after laparoscopic gynecological surgery: A randomized controlled trial. | Gungorduk et al | 2020 |
| Dexamethasone on postoperative gastrointestinal motility: A placebo-controlled, double-blinded, randomized controlled trial. | Chen et al | 2020 |
| Neuromuscular blockade reversal with sugammadex versus pyridostigmine/ glycopyrrolate in laparoscopic cholecystectomy: A randomized trial of effects on postoperative gastrointestinal motility. | An et al | 2020 |
| The impact of chewing gum on postoperative bowel activity and postoperative pain after total laparoscopic hysterectomy. | Turkay et al | 2020 |
| Randomized controlled trial to compare outcomes with and without the enhanced recovery after surgery protocol in patients undergoing radical cystectomy. | Bansal et al | 2020 |
| Efficacy of chewing gum for improving bowel function after cesarean sections: a randomized controlled trial. | Yenigul et al | 2020 |
| Comparison of enhanced recovery protocol with conventional care in patients undergoing minor gynecologic surgery. | Yilmaz et al | 2020 |
| Randomized, Double-Blind Study of the Effect of Intraoperative Intravenous Lidocaine on the Opioid Consumption and Criteria for Hospital Discharge After Bariatric Surgery. | Sakata et al. | 2020 |
| The effect of gum chewing on the return of bowel motility after planned cesarean delivery: a randomized controlled trial. | Altraigey et al | 2020 |
| Dexmedetomidine combined with ropivacaine in ultrasound-guided tranversus abdominis plane block improves postoperative analgesia and recovery following laparoscopic colectomy. | Weizhong et al | 2020 |
| Goal-directed versus Standard Fluid Therapy to Decrease Ileus after Open Radical Cystectomy: A Prospective Randomized Controlled Trial. | Arslan-Carlon et al | 2020 |
| The effects of carum carvi (Bunium persicum boiss) on early return of bowel motility after caesarean section: Double-blind, randomized, placebo-controlled trial. | Yousefi et al | 2019 |
| Effects of early oral feeding versus delayed feeding on gastrointestinal function of post-caesarean section women in a tertiary hospital in Enugu, Nigeria: A randomized controlled trial. | Mba et al | 2019 |
| Effects of Cassia alata Linn on bowel function recovery following surgery for gynecological cancer: A randomized controlled trial. | Phutsisen et al. | 2019 |
| Effects of a metabolic optimized fast track concept (MOFA) on bowel function and recovery after surgery in patients undergoing elective colon or liver resection: A randomized controlled trial. | Uhlig et al | 2019 |
| The effects of Carumcarvi (Buniumpersicumboiss) on early return of bowel motility after caesarean section: Double-blind, randomized, placebo-controlled trial. | Yousefi et al | 2019 |
| Quantification of the effects of coffee on postoperative ileus after laparoscopic ventral rectopexy: a randomized controlled trial. | Hayashi et al | 2019 |
| Does Coffee Intake Reduce Postoperative Ileus after Laparoscopic Elective Colorectal Surgery? A Prospective, Randomized Controlled Study: The Coffee Study. | Hasler-Gehrer et al | 2019 |
| Impact of pre-operative walking on post-operative bowel function in patients with gynecologic cancer. | Ozdemir et al | 2019 |
| Chewing aid in routine postoperative orders-does it reduce postoperative ileus after cesarean section? A randomized control trial. | Ali et al | 2019 |
| Usage of intravenous lidocaine infusion with enhanced recovery pathway in patients scheduled for open radical cystectomy: A randomized trial. | Moeen and Moeen | 2019 |
| Effect of alvimopan on gastrointestinal recovery and length of hospital stay after retroperitoneal lymph node dissection for testicular cancer. | Radadia et al | 2019 |
| Evaluation of the effect of oral pyridostigmine on the ileus after abdominal surgery: A blinded randomized clinical trial. | Maleknejad et al | 2018 |
| Intravenous lidocaine infusions for 48 hours in open colorectal surgery: A prospective, randomized, double-blinded, placebo-controlled trial. | Ho et al | 2018 |
| Multicentre randomized clinical trial of the effect of chewing gum after abdominal surgery. | de Leede et al | 2018 |
| A randomized, double-blind, placebo-controlled trial on the efficacy of ginger in the prevention of abdominal distention in post cesarean section patients. | Tianthong et al | 2018 |
| Randomized controlled trial study for evaluation of herbal lozenge compared with standard postoperative care to enhance postoperative bowel function recovery following elective open abdominal surgery. | Lertbannaphonet al | 2018 |
| Perioperative transcutaneous tibial nerve stimulation to reduce postoperative ileus after colorectal resection: A pilot study. | Venara et al | 2018 |
| Gum chewing aids bowel function return and analgesic requirements after bowel surgery: a randomized controlled trial. | Byrne et al | 2018 |
| Continuous administration of ramosetron with patient-controlled analgesia after laparoscopic distal gastrectomy does not delay postoperative bowel function recovery: A prospective, randomized, double-blinded study. | Jung et al | 2018 |
| The effect of simethicone on postoperative ileus in patients undergoing colorectal surgery (SPOT), a randomized controlled trial. | Springer et al | 2018 |
| Chewing xylitol gum could accelerate bowel motility recovery after elective open proctectomy for rectal cancer. | Yang et al | 2018 |
| Perioperative lipid-enriched enteral nutrition versus standard care in patients undergoing elective colorectal surgery (SANICS II): a multicentre, double-blind, randomised controlled trial. | Peters et al | 2018 |
| Effect of topical chamomile oil on postoperative bowel activity after cesarean section: A randomized controlled trial. | Khadem et al | 2018 |
| Comparison of mean time of postoperative return of bowel movements with or without nasogastric tube in elective abdominal surgeries. | Haroon et al | 2018 |
| Effect of Acupressure on Symptoms of Postoperative Ileus After Cesarean Section. | Shahabinejad et al | 2017 |
| Thoracic epidural versus intravenous patient-controlled analgesia after open colorectal cancer surgery. | Radovanovic et al. | 2017 |
| Nicotine chewing gum for the prevention of postoperative ileus after colorectal surgery: a multicenter, double-blind, randomised, controlled pilot study. | Lambrichts et al | 2017 |
| The Role of Transversus Abdominis Plane Blocks in Enhanced Recovery after Surgery Pathways for Open and Laparoscopic Colorectal Surgery. | Kim et al | 2017 |
| Comparison of the effects of patient-controlled epidural and intravenous analgesia on postoperative bowel function after laparoscopic gastrectomy: a prospective randomized study. | Cho et al | 2017 |
| Comparison of multi-modal early oral nutrition for the tolerance of oral nutrition with conventional care after major abdominal surgery: A prospective, randomized, single-blind trial. | Sun et al | 2017 |
| Goal-directed Fluid Therapy Does Not Reduce Primary Postoperative Ileus after Elective Laparoscopic Colorectal Surgery: A Randomized Controlled Trial. | Gomez-Izquierdo et al | 2017 |
| Crystalloid versus colloid fluids for reduction of postoperative ileus after abdominal operation under combined general and epidural anesthesia. | Ghodraty et al | 2017 |
| Effect of gum chewing and coffee consumption on intestinal motility in caesarean sections. | Goymen et al | 2017 |
| Effectiveness of continuous wound infusion of local anesthetics after abdominal surgeries. | Sistla et al | 2017 |
| Effects of coffee consumption on gut recovery after surgery of gynecological cancer patients: a randomized controlled trial. | Gungorduk et al | 2017 |
| Effect of acupuncture on postoperative ileus after distal gastrectomy for gastric cancer. | Jung et al | 2017 |
| Comparison of efficacy of simo decoction and acupuncture or chewing gum alone on postoperative ileus in colorectal cancer resection: a randomized trial. | Yang et al | 2017 |
| Intraoperative continuous intestinal loop warming technique A prospective randomised trial. | Rulli et al | 2017 |
| Gum chewing combined with oral intake of a semi-liquid diet in the postoperative care of patients after gynaecologic laparoscopic surgery. | Pan et al | 2017 |
| Influence of gum-chewing on postoperative bowel activity after laparoscopic surgery for gastric cancer: A randomized controlled trial. | Ge et al | 2017 |
| Effect of chewing gum on recovery of gastrointestinal function after splenectomy and pericardial devascularization. [Chinese] | Liu et al | 2016 |
| Evaluation of the efficacy of daikenchuto (TJ -100) for the prevention of paralytic ileus after pancreaticoduodenectomy: A multicenter, double-blind, randomized, placebo-controlled trial. | Okada et al | 2016 |
| Effect of sham feeding with gum chewing on postoperative ileus after liver transplantation-a randomized controlled trial. | Lee et al. | 2016 |
| Effect of acupuncture on reducing duration of postoperative ileus after gastrectomy in patients with gastric cancer: A pilot study using sitz marker. | Chae et al | 2016 |
| The effect of perioperative probiotics treatment for colorectal cancer: Short-term outcomes of a randomized controlled trial. | Yang et al | 2016 |
| Clinical pharmacology of daikenchuto assessed by transit analysis using radiopaque markers in patients with colon cancer undergoing open surgery: a multicenter double-blind randomized placebo-controlled study (JFMC39-0902 additional study). | Katsuno et al | 2016 |
| Randomised clinical trial: Prucalopride, a colonic pro-motility agent, reduces the duration of post-operative ileus after elective gastrointestinal surgery. | Gong et al | 2016 |
| Early versus Late Feeding after Cesarean Delivery: A Randomized Controlled Trial. | Saad et al | 2016 |
| Effect of gum chewing in the reduction of paralytic ileus following cholecystectomy. | Pilevarzadeh M. | 2016 |
| Extracorporeal vs. intracorporeal ileocolic stapled anastomoses in laparoscopic right colectomy: An interim analysis of a randomized clinical trial. | Vignali et al | 2016 |
| Reversal of neuromuscular blockade with sugammadex or neostigmine/atropine: Effect on postoperative gastrointestinal motility. | Sen et al | 2016 |
| Effect of conventional diet schedule, early feeding and early feeding plus domperidone on postcesarean diet tolerance: A randomized controlled trial. | Nantasupha et al. | 2016 |
| Dexmedetomidine improves gastrointestinal motility after laparoscopic resection of colorectal cancer: A randomized clinical trial. | Chen et al | 2016 |
| Influence of gum chewing on the recovery of gastrointestinal function in patients undergoing abdominal complete staging surgery for gynecological malignancies. [Chinese] | Li et al | 2016 |
| Effect of gum chewing on bowel function recovery in patients after radical cystectomy with urinary diversion. [Chinese] | Wang et al | 2016 |
| Usefulness of Gum Chewing to Decrease Postoperative Ileus in Colorectal Surgery with Primary Anastomosis: A Randomized Controlled Trial. | Vergara-Fernandez et al | 2016 |
| Effect of gum chewing on reducing postoperative ileus and recovery after colorectal surgery: A randomised controlled trial. | Topcu and Oztekin | 2016 |
| The Effect of Warm Water Intake on Bowel Movements in the Early Postoperative Stage of Patients Having Undergone Laparoscopic Cholecystectomy: A Randomized Controlled Trial. | Caliskan et al | 2016 |
| Effect of bed exercises and gum chewing on abdominal sounds, flatulence and early discharge in the early period after caesarean section. | Cevik and Baser | 2016 |
| Randomized clinical trial of postoperative chewing gum versus standard care after colorectal resection. | Atkinson et al | 2016 |
| The Role of Xylitol Gum Chewing in Restoring Postoperative Bowel Activity After Cesarean Section. | Lee et al | 2016 |
| A Double-Blinded Randomized Clinical Study on the Therapeutic Effect of Gastrografin in Prolonged Postoperative Ileus After Elective Colorectal Surgery. | Biondo et al | 2016 |
| Metoclopramide role in preventing ileus after cesarean, a clinical trial. | Agah et al | 2015 |
| Gum chewing reduces the time to first defaecation after pelvic surgery: A randomised controlled study. | Tazegul et al | 2015 |
| Xylitol Gum Chewing to Achieve Early Postoperative Restoration of Bowel Motility after Laparoscopic Surgery. | Gong et al | 2015 |
| Efficacy of gum chewing on bowel movement after open colectomy for left-sided colorectal cancer: A randomized clinical trial. | Kobayashi et al | 2015 |
| Effect of coffee on the length of postoperative ileus after elective laparoscopic left-sided colectomy: A randomized, prospective single-center study. | Dulskas et al | 2015 |
| Perioperative administration of daikenchuto (TJ-100) reduces the postoperative paralytic ileus in patients with pancreaticoduodenectomy. | Okada et al | 2015 |
| Gastrografin in prolonged postoperative ileus: A double-blinded randomized controlled trial. | Vather et al | 2015 |
| Effect of Daikenchuto, a Traditional Japanese Herbal Medicine, after Total Gastrectomy for Gastric Cancer: A Multicenter, Randomized, Double-Blind, Placebo-Controlled, Phase II Trial. | Yoshikawa et al | 2015 |
| The Effects of Drinking Coffee While Recovering from Colon and Rectal Resection Surgery. | Piric et al | 2015 |
| Efficacy and safety of low-dose celecoxib in reducing post-operative paralytic ileus after major abdominal surgery. | Raju et al | 2015 |
| Effects of daikenchuto, a Japanese herb, on intestinal motility after total gastrectomy: a prospective randomized trial. | Akamaru et al | 2015 |
| The Effect of Gum Chewing, Early Oral Hydration, and Early Mobilization on Intestinal Motility After Cesarean Birth. | Sahin and Terzioglu | 2015 |
| The Effect of Fennel Tea Drinking on Postoperative Gut Recovery after Gynecological Malignancies Operation. [Chinese] | Ma et al | 2015 |
| Effects of chewing gum against postoperative ileus after pancreaticoduodenectomy--a randomized controlled trial. | Andersson et al | 2015 |
| Effect of intravenous lignocaine on perioperative stress response and post-surgical ileus in elective open abdominal surgeries: a double-blind randomized controlled trial. | Sridhar et al | 2015 |
| Randomized clinical trial of the effect of gum chewing on postoperative ileus and inflammation in colorectal surgery. | van den Heijkant et al | 2015 |
| Alvimopan accelerates gastrointestinal recovery after radical cystectomy: A multicenter randomized placebo-controlled trial. | Lee et al | 2014 |
| Reduction of postoperative ileus by early enteral nutrition in patients undergoing major rectal surgery: Prospective, randomized, controlled trial. | Boelens et al | 2014 |
| Fast-track versus standard care in laparoscopic high anterior resection: A prospective randomized-controlled trial. | Mari et al | 2014 |
| Prospective, randomized, controlled, proof-of-concept study of the Ghrelin mimetic ipamorelin for the management of postoperative ileus in bowel resection patients. | Beck et al | 2014 |
| Stimulation of the efferent limb before ileostomy closure: A randomized clinical trial. | Abrisqueta et al | 2014 |
| Early use of low residue diet is superior to clear liquid diet after elective colorectal surgery a randomized controlled trial. | Lau et al | 2014 |
| The effect of the gelatinous lactulose for postoperative bowel movement in the patients undergoing cesarean section. | Shigemi et al | 2014 |
| The efficacy of chewing gum on postoperative ileus following cesarean section in Enugu, South East Nigeria: A randomized controlled clinical trial. | Ajuzieogu et al | 2014 |
| Effects of intravenous versus epidural lidocaine infusion on pain intensity and bowel function after major large bowel surgery: a double-blind randomized controlled trial. | Staikou C. | 2014 |
| Electroacupuncture at ST36 accelerates the recovery of gastrointestinal motility after colorectal surgery: a randomised controlled trial. | Zhang et al | 2014 |
| Multi-institutional outcomes and cost effectiveness of using alvimopan to lower gastrointestinal morbidity after cystectomy and urinary diversion. | Vora et al | 2014 |
| Early oral feeding following laparoscopic colorectal cancer surgery. | Kim et al | 2014 |
| The efficacy of gum chewing in reducing postoperative ileus: a multisite randomized controlled trial. | Forrester et al | 2014 |
| Evaluating the efficacy of current treatments for reducing postoperative ileus: A randomized clinical trial in a single center. | Bonventre et al | 2014 |
| Daikenchuto stimulates colonic motility after laparoscopic-assisted colectomy. | Yaegashi et al | 2014 |
| Early oral feeding vs. traditional feeding in patients undergoing elective open bowel surgery-a randomized controlled trial. | Pragatheeswarane et al | 2014 |
| Electroacupuncture reduces duration of postoperative ileus after laparoscopic surgery for colorectal cancer. | Ng et al | 2013 |
| Sham feeding with chewing gum after elective colorectal resectional surgery: A randomized clinical trial. | Lim et al | 2013 |
| Effect of Daikenchuto (TJ-100) on postoperative bowel motility and on prevention of paralytic ileus after pancreaticoduodenectomy: A multicenter, randomized, placebo-controlled phase II trial (The Japan-PD study). | Okada et al | 2013 |
| Postoperative gum chewing after gynecologic laparoscopic surgery: A randomized controlled trial. | Husslein et al | 2013 |
| Safety and efficacy of ulimorelin administered postoperatively to accelerate recovery of gastrointestinal motility following partial bowel resection: Results of two randomized, placebo-controlled phase 3 trials. | Shaw et al | 2013 |
| Prophylactic nasogastric decompression after emergency laparotomy. | Sapkota and Bhandari | 2013 |
| Chewing sugar-free gum reduces ileus after cesarean section in nulliparous women: A randomized clinical trial. | Mohsenzadeh et al | 2013 |
| A prospective randomized controlled trial of sugared chewing gum on gastrointestinal recovery after major colorectal surgery in patients managed with early enteral feeding. | Zaghiyan et al | 2013 |
| Influence of gum chewing on postoperative bowel activity after complete staging surgery for gynecological malignancies: A randomized controlled trial. | Ertas et al | 2013 |
| A phase II, randomized, controlled trial of acupuncture for reduction of postcolectomy ileus. | Deng et al | 2013 |
| Electroacupuncture for ileus after laparoscopic colorectal surgery: a randomised sham-controlled study. | Ng et al | 2013 |
| Multimodal interventions (chewing gum, early oral hydration and early mobilisation) on the intestinal motility following abdominal gynaecologic surgery. | Terzioglu et al | 2013 |
| The beneficial effect of ST-36 (Zusanli) acupressure on postoperative gastrointestinal function in patients with colorectal cancer. | Chao et al | 2013 |
| Post-operative ileus in hemicolectomy for cancer: open versus laparoscopic approach. | Procacciante et al | 2013 |
| Effect of gum chewing after cesarean-delivery on return of bowel function. [Persian] | Mohsenzadeh et al | 2012 |
| Effect of intraoperative intravenous lidocaine on postoperative pain and return of bowel function after laparoscopic abdominal gynecologic procedures. | Grady et al | 2012 |
| Bowel function after minimally invasive urogynecologic surgery: a prospective randomized controlled trial. | McNanley et al | 2012 |
| The beneficial effects of Kampo medicine Dai-Ken-Chu-To after hepatic resection: A prospective randomized control study. | Nishi et al | 2012 |
| Role of gum chewing on the duration of postoperative ileus following ileostomy closure done for typhoid ileal perforation: A prospective randomized trial. | Marwah et al | 2012 |
| Effect of the laxative magnesium oxide on gastrointestinal functional recovery in fast-track colonic resection: A double-blind, placebo-controlled randomized study. | Andersen et al | 2012 |
| Chewing gums has stimulatory eff ects on bowel function in patients undergoing cesarean section: A randomized controlled trial. | Ledari et al | 2012 |
| Evaluation of the effect of intera-peritoneal bupivacaine on post operative bowel motility and analgesia. [Persian] | Boeni et al | 2012 |
| Alvimopan provides rapid gastrointestinal recovery without nasogastric tube decompression after radical cystectomy and urinary diversion. | Vora et al | 2012 |
| [Adhesive ileus treated by electroacupuncture at Zhigou (TE 6) and Zusanli (ST 36): a randomized controlled study]. [Chinese] | Wen et al | 2012 |
| Effects of glutamine on gastrointestinal motor activity in patients following gastric surgery. | Mochiki et al | 2011 |
| Safety and efficacy of methylnaltrexone in shortening the duration of postoperative ileus following segmental colectomy: Results of two randomized, placebo-controlled phase 3 trials. | Yu et al | 2011 |
| Intravenous lidocaine versus thoracic epidural analgesia: A randomized controlled trial in patients undergoing laparoscopic colorectal surgery using an enhanced recovery program. | Wongyingsinn et al | 2011 |
| A double-blind randomized controlled clinical trial to assess the effect of doppler optimized intraoperative fluid management on outcome following radical cystectomy. | Pillai et al | 2011 |
| Treatment of postoperative ileus with choline citrate-results of a prospective, randomised, placebo-controlled, double-blind multicentre trial. | Herzog et al | 2011 |
| A simplified rehabilitation program for patients undergoing elective colonic surgery - Randomized controlled clinical trial. | Da Fonseca et al | 2011 |
| A randomized, placebo-controlled phase 3 trial (study sb-767905/013) of alvimopan for opioid-induced bowel dysfunction in patients with non-cancer pain. | Irving et al | 2011 |
| Chewing gum has a stimulatory effect on bowel motility in patients after open or robotic radical cystectomy for bladder cancer: A prospective randomized comparative study. | Choi et al | 2011 |
| Intravenous lidocaine is as effective as epidural bupivacaine in reducing ileus duration, hospital stay, and pain after open colon resection: A randomized clinical trial. | Swenson et al | 2010 |
| Chewing gum reduces postoperative ileus after open appendectomy. | Ngowe et al | 2010 |
| The ghrelin agonist TZP-101 for management of postoperative ileus after partial colectomy: A randomized, dose-ranging, placebo-controlled clinical trial. | Popescu et al | 2010 |
| Gum chewing slightly enhances early recovery from postoperative Ileus after cesarean section: Results of a prospective, randomized, controlled trial. | Shang et al | 2010 |
| Wound infusion with local anaesthesia after laparotomy: A randomized controlled trial. | Wang et al | 2010 |
| A randomized trial of rocking-chair motion on the effect of postoperative ileus duration in patients with cancer recovering from abdominal surgery. | Massey R.L. | 2010 |
| Randomized clinical trial of laxatives and oral nutritional supplements within an enhanced recovery after surgery protocol following liver resection. | Hendry et al | 2010 |
| Influence of gum chewing on postoperative bowel activity after cesarean section. | Kafali et al | 2010 |
| Implementing fast-track protocol for colorectal surgery: A prospective randomized clinical trial. | Ionescu et al | 2009 |
| Opioid-sparing effects of ketorolac and its correlation with the recovery of postoperative bowel function in colorectal surgery patients: A prospective randomized double-blinded study. | Chen et al | 2009 |
| A randomised controlled trial of early initiation of oral feeding after cesarean section. | Orji et al | 2009 |
| Gum chewing stimulates early return of bowel motility after caesarean section. | Abd-El-Maeboud et al | 2009 |
| Clinical trial: The impact of cyclooxygenase inhibitors on gastrointestinal recovery after major surgery - A randomized double blind controlled trial of celecoxib or diclofenac vs. placebo. | Wattchow et al | 2009 |
| Pilot postoperative ileus study of escin in cancer patients after colorectal surgery. | Xie et al | 2009 |
| Early oral hydration after cesarean delivery performed under regional anesthesia. | Mulayim et al | 2008 |
| A randomised controlled trial on beneficial effects of early feeding post-Caesarean delivery under regional anaesthesia. | Shah et al | 2007 |
| Effect of mosapride on recovery of intestinal motility after hand-assisted laparoscopic colectomy for carcinoma. | Narita et al | 2008 |
| Gastrointestinal tract recovery in patients undergoing bowel resection: Results of a randomized trial of alvimopan and placebo with a standardized accelerated postoperative care pathway. | Ludwig et al | 2008 |
| Acupuncture to prevent prolonged postoperative ileus: A randomized controlled trial. | Garcia et al | 2008 |
| Effect of bisacodyl on postoperative bowel motility in elective colorectal surgery: A prospective, randomized trial. | Zingg et al | 2008 |
| Neostigmine in unavoidable post operative ileus: A randomized clinical trial. | Fanaei et al | 2008 |
| Clinical trial: Alvimopan for the management of post-operative ileus after abdominal surgery: results of an international randomized, double-blind, multicentre, placebo-controlled clinical study. | Buchler et al | 2008 |
| A prospective randomized double-blind study to determine the effect of thoracic epidural neostigmine on postoperative ileus after abdominal aortic surgery. | Caliskan et al | 2008 |
| Intravenous flurbiprofen axetil accelerates restoration of bowel function after colorectal surgery. | Xu et al | 2008 |
| Prospective randomized, double-blind, placebo-controlled study of pre- and postoperative administration of a COX-2-specific inhibitor as opioid-sparing analgesia in major colorectal surgery. | Sim et al | 2007 |
| Bowel function after bowel surgery: Morphine with ketamine or placebo; a randomized controlled trial pilot study. | McKay and Donais | 2007 |
| Systemic lidocaine shortens length of hospital stay after colorectal surgery: A double-blinded, randomized, placebo-controlled trial. | Herroeder et al | 2007 |
| Thoracic epidural analgesia facilitates the restoration of bowel function and dietary intake in patients undergoing laparoscopic colon resection using a traditional, nonaccelerated, perioperative care program. | Taqi et al | 2007 |
| Treatment of Postoperative Ileus After Bowel Surgery with Low-Dose Intravenous Erythromycin. | Lightfoot et al | 2007 |
| Optimizing recovery after laparoscopic colon surgery (ORAL-CS): Effect of intravenous Ketorolac on length of hospital stay. | Schlachta et al | 2007 |
| Early removing gastrointestinal decompression and early oral feeding improve patients' rehabilitation after colorectostomy. | Zhou et al | 2006 |
| Alvimopan, a peripherally acting mu-opioid receptor antagonist, compared with placebo in postoperative ileus after major abdominal surgery: Results of a randomized, double-blind, controlled study. | Viscusi et al | 2006 |
| Does gum chewing reduce postoperative ileus following open colectomy for left-sided colon and rectal cancer? - A prospective randomized controlled trial. | Quah et al | 2006 |
| A double-blind, randomized, placebo-controlled phase III study of the safety of alvimopan in patients who undergo simple total abdominal hysterectomy. | Herzog et al | 2006 |
| The duration of postoperative ileus after elective colectomy is correlated to surgical specialization. | Gervaz et al | 2006 |
| Gum chewing reduces ileus after elective open sigmoid colectomy. | Schuster et al | 2006 |
| Does Gum Chewing Ameliorate Postoperative Ileus? Results of a Prospective, Randomized, Placebo-Controlled Trial. | Matros et al | 2006 |
| Pre-operative oral carbohydrate loading in colorectal surgery: A randomized controlled trial. | Noblett et al | 2006 |
| Early oral feeding should be resumed following the resolution of gastric ileus. | Tsunoda et al | 2005 |
| Preincisional dextromethorphan combined with thoracic epidural anesthesia and analgesia improves postoperative pain and bowel function in patients undergoing colonic surgery. | Yeh et al | 2005 |
| Effect of adding ketorolac to intravenous morphine patient-controlled analgesia on bowel function in colorectal surgery patients - A prospective, randomized, double-blind study. | Chen et al | 2005 |
| Effect of bupivacaine after operative laparoscopic gynecologic procedures. | Ceyhan et al | 2005 |
| Alvimopan, a novel, peripherally acting mu opioid antagonist: Results of a multicenter, randomized, double-blind, placebo-controlled, phase III trial of major abdominal surgery and postoperative ileus. | Wolff et al | 2004 |
| The effect of epidural clonidine on perioperative cytokine response, postoperative pain, and bowel function in patients undergoing colorectal surgery. | Wu et al | 2004 |
| Randomized clinical trial of itopride for the treatment of postoperative ileus after laparoscopic cholecystectomy. | Gurlich et al | 2004 |
| The Effects of Combined Gum-chewing and Parenteral Metoclopramide on the Duration of Postoperative Ileus After Abdominal Surgery. | Ulasi et al | 2023 |
| The Effect of Chewing Sugar-Free Gum to Improve Bowel Movement After Colorectal Surgeries in Patients With Colorectal Cancer. | Al-Kharabsheh et al | 2023 |
| The Effect of Coffee Consumption after Colorectal Surgery on Bowel Movements and Duration of Hospital Stay. | Bildirici et al | 2023 |
| Effects of Nonsteroidal Anti-Inflammatory Drugs as Patient Controlled Analgesia on Early Bowel Function Recovery after Radical Cystectomy. | Yu et al | 2018 |
| [Effect of Modified Dachengqi Decoction on Recovery of Gastrointestinal Function after Surgery in Patients with Rectal Cancer]. [Chinese] | Yu et al | 2017 |
| Early Versus Delayed Oral Feeding after Uncomplicated Cesarean Section under Spinal Anesthesia: A Randomized Controlled Trial. | Ogbadua et al | 2018 |
| Randomized clinical trial of chewing gum after laparoscopic colorectal resection. | Shum et al | 2016 |
| Randomized Clinical Trial Comparing Efficacy of Simo Decoction and Acupuncture or Chewing Gum Alone on Postoperative Ileus in Patients With Hepatocellular Carcinoma After Hepatectomy. | You et al | 2015 |
| Clinical efficacy of Daikenchuto for gastrointestinal dysfunction following colon surgery: a randomized, double-blind, multicenter, placebo-controlled study (JFMC39-0902). | Katsuno et al | 2015 |
| Ringer's lactate, but not hydroxyethyl starch, prolongs the food intolerance time after major abdominal surgery; an open-labelled clinical trial. | Li et al | 2015 |
| Gum chewing promotes bowel motility after a radical retropubic prostatectomy. | Choi et al | 2014 |
| Effect of polylactic film (Surgi-Wrap) on preventing postoperative ileus after major hepato-pancreato-biliary surgery. | Lee et al | 2016 |
| Magnesium Can Decrease Postoperative Physiological Ileus and Postoperative Pain in Major non Laparoscopic Gastrointestinal Surgeries: A Randomized Controlled Trial. | Shariat et al | 2014 |
| Randomized placebo-controlled study of intravenous methylnaltrexone in postoperative ileus. | Viscusi et al | 2013 |
| Gum chewing enhances early recovery of bowel function following transperitoneal abdominal aortic surgery. | Takagi et al | 2012 |
| Is lidocaine patch as effective as intravenous lidocaine in pain and illus reduction after laparoscopic colorectal surgery? A randomized clinical trial. | Elhafz et al | 2012 |
| Mosapride citrate improves postoperative ileus of patients with colectomy. | Toyomasu et al | 2011 |
| Early removal of nasogastric tube is beneficial for patients undergoing radical cystectomy with urinary diversion. | Adamakis et al | 2011 |
| [Usefulness of chewing gum for the resolution of postoperative ileus in left colon resections]. [Spanish] | Bahena-Aponte et al | 2010 |
| Comparing early oral feeding with traditional oral feeding in upper gastrointestinal surgery. | Hosseini et al | 2010 |
| Electro-acupuncture to prevent prolonged postoperative ileus: a randomized clinical trial. | Meng et al | 2010 |
| Comparison of methods to facilitate postoperative bowel function. | Crainic et al | 2009 |
| Gum chewing stimulates bowel motility in patients undergoing radical cystectomy with urinary diversion. | Kouba et al | 2007 |
| Effect of laxatives on gastrointestinal functional recovery in fast-track hysterectomy: a double-blind, placebo-controlled randomized study. | Hansen et al | 2007 |
| A randomised controlled trial on beneficial effects of early feeding post-Caesarean delivery under regional anaesthesia. | Teoh et al | 2007 |
| Effect of Simotang oral liquid on anal exhaust in patients after abdominal gynecological operation. | Chen and Wang | 2006 |
| Laparoscopy for colectomy accelerates restoration of bowel function when using patient controlled analgesia. | Hong et al | 2006 |
| Gum-chewing stimulates bowel motility after surgery for colorectal cancer. | Hirayama et al | 2006 |
| Preventing prolonged post-operative ileus in gastric cancer patients undergoing gastrectomy and intra-peritoneal chemotherapy. | Chan et al | 2005 |
| Phase III trial of alvimopan, a novel, peripherally acting, mu opioid antagonist, for postoperative ileus after major abdominal surgery. | Delaney et al | 2005 |
| The effect of the herbal medicines dai-kenchu-to and keishi-bukuryo-gan on bowel movement after colorectal surgery. | Suehiro et al | 2005 |
